# Experimental inflammation following dural application of complete Freund’s adjuvant or inflammatory soup does not alter brain and trigeminal microvascular passage

**DOI:** 10.1186/s10194-015-0575-8

**Published:** 2015-10-28

**Authors:** Cornelia Lundblad, Kristian A. Haanes, Gustaf Grände, Lars Edvinsson

**Affiliations:** Department of Medicine, Institute of Clinical Sciences, University Hospital, Lund University, 22185 Lund, Sweden; Department of Clinical Experimental Research, Copenhagen University Hospital, Glostrup, Denmark

**Keywords:** Microvascular passage, Transfer constant, Trigeminal ganglion, Inflammatory soup, Complete Freund’s Adjuvant

## Abstract

**Background:**

Migraine is a paroxysmal, disabling primary headache that affects 16 % of the adult population. In spite of decades of intense research, the origin and the pathophysiology mechanisms involved are still not fully known. Although triptans and gepants provide effective relief from acute migraine for many patients, their site of action remains unidentified. It has been suggested that during migraine attacks the leakiness of the blood-brain barrier (BBB) is altered, increasing the passage of anti-migraine drugs. This study aimed to investigate the effect of experimental inflammation, following dural application of complete Freund’s adjuvant (CFA) or inflammatory soup (IS) on brain and trigeminal microvascular passage.

**Methods:**

In order to address this issue, we induced local inflammation in male Sprague-Dawley-rats dura mater by the addition of CFA or IS directly on the dural surface. Following 2, 24 or 48 h of inflammation we calculated permeability-surface area product (PS) for [^51^Cr]-EDTA in the trigeminal ganglion (TG), spinal trigeminal nucleus, cortex, periaqueductal grey and cerebellum.

**Results:**

We observed that [^51^Cr]-EDTA did not pass into the central nervous system (CNS) in a major way. However, [^51^Cr]-EDTA readily passed the TG by >30 times compared to the CNS. Application of CFA or IS did not show altered transfer constants.

**Conclusions:**

With these experiments we show that dural IS/CFA triggered TG inflammation, did not increase the BBB passage, and that the TG is readily exposed to circulating molecules. The TG could provide a site of anti-migraine drug interaction with effect on the trigeminal system.

**Electronic supplementary material:**

The online version of this article (doi:10.1186/s10194-015-0575-8) contains supplementary material, which is available to authorized users.

## Background

Migraine is a neurological disorder that afflicts up to 16 % of the adult population in the Western countries [1]. It is episodic, often with moderate to severe disabling headache, associated with sensory and autonomic symptoms, phonophobia and photophobia, and cognitive symptoms. Many researchers consider migraine to be a disorder in which Central Nervous System (CNS) dysfunction plays a pivotal role while various parts of the trigeminovascular system are necessary for the expression of peripheral symptoms and aspects of pain [2].

Several drugs have been shown to reduce migraine symptoms, or elicits migraine-like attacks [3]. Studies, both in man and in experimental animals, have revealed anti-migraine effects of triptans, gepants (calcitonin gene-related peptide (CGRP) antagonists) and CGRP antibodies [4–7]. However, due to the size and the pharmacokinetics of these drugs it is suggested that only a modest passage may occur across the blood-brain barrier (BBB) [8]. The published figures on BBB passage are in the vicinity of 3 % when given at clinically effective doses of triptans/gepants [5, 9–11]. While these molecules have a small size, the CGRP antibodies will not likely cross the BBB at all and therefore unlikely to antagonize receptor sites within the CNS [5].

In order to neutralize this argument some researchers have suggested that the BBB is modified during migraine attacks and may show enhanced passage [12]. From a purely mechanistic viewpoint the BBB consists of a myriad of molecules, including metalloproteases, as well as claudins and integrins which would negate a dynamic opening of the closed tight junctions which are an essential part of the BBB [13, 14]. Another aspect that must be considered, that has not been proven, is modification of transport guided passage across the BBB which could be modified during the attacks.

Therefore, it is possible that candidates for targets of migraine treatment may reside outside the BBB; the dural vasculature, the trigeminal ganglion (TG) and in CNS regions lacking a BBB such as pituitary and pineal glands [15] and at nerve afferents that connect to the TG. The dural vasculature, including the middle meningeal artery (MMA) has long been considered as a target based on the vasogenic theory of migraine [16, 17]. We and others have shown that both sumatriptan and telcagepant can inhibit CGRP induced vasodilation in the human MMA, illustrating one of its potential targets [18, 19]. However, not all vasodilators cause migraine and triptans are not effective in all migraine patients [20].

The other possibility is the activation and involvement of the TG with its afferents which innervates the dura mater and the MMA [21]. Based on preceding studies on TG cell [22] and TG organ cultures [23], we have recently shown that the addition of complete Freund’s adjuvant (CFA) or inflammatory soup (IS) on the dura mater causes an increase in phosphorylated extracellular signal-regulated kinases (p-ERK1/2) activation and interleukin (IL)-1β expression in the ipsilateral TG [24]. The application of inflammatory substances on the dura mater, in addition to chemical stimulation of the dural receptive fields, cause hypersensitivity to mechanical and thermal stimulation, as well as activation in the TG and in brain stem trigeminal neurons, similar to what could be expected in migraine [17, 24]. BBB permeability has never been investigated using this model. It is highly relevant to ask this question as it has been suggested that the BBB might change during a migraine attack in effort to explain how anti-migraine drugs can reach the CNS [12, 25–28]. In addition, it is relevant to compare the permeability to the TG and evaluate any potential alterations.

Here we report that there are no differences in the permeability into the CNS after experimental inflammation and there are no significant changes in brain water content or in permeability. Interestingly, we show that the TG is more than 30 times more permeable than the CNS at normal conditions and thereby supporting the idea that anti-migraine drugs may reach much higher and clinically relevant levels within the TG than they do in the CNS.

## Methods

### Animals

The experimental protocol was approved by the local Ethical Committee for Animal Research (M38-13). Adult male Sprague-Dawley rats (*n* = 35) (Taconic Copenhagen, Denmark) were used to investigate the brain water content and the permeability-surface area product (PS) for [^51^Cr]-EDTA. There was a reversed 12–12 light cycle, with darkness during the day. The animals were treated in accordance with the national guidelines for laboratory animals (publications DFS 2004:4, the Swedish Board of Agriculture)

The animals were anesthetized by intraperitoneal injection of sodium pentobarbital (60 mg/kg, Pentobarbitalnatrium, vet.APL, 60 mg/ml, APL, Stockholm) and then placed on a heating pad and in a stereotactic head holder. Body core temperature was kept at 37 °C by a feedback circuit controlled by continuous measurement of rectal temperature. A parasagittal craniotomy, with a diameter of 6 mm, was performed in the left parietal bone between the coronal and lambdoid sutures with the center approximately 5 mm from the coronal suture and 5 mm from the sagittal suture, after a scalp incision. The animals were randomly assigned to three different solutions applied to the surface of the exposed dura in a volume of 10 μL; i) vehicle (saline), ii) CFA used as stock solution (Sigma Aldrich, Life Science, US), and iii) an IS. The recipe of IS was adapted from Strassman and colleagues [29] and contains 10 μM each of bradykinin, serotonin and prostaglandin E2, 100 μM histamine at a pH of 5.0 (all obtained from Sigma Aldrich). After 15 min CFA or IS was removed by flushing with saline. Surgical staples were used to close the scalp incision and the animals were moved to another heating pad for recovering. At 2, 24 or 48 h following application to the dura, the animals were re-anesthetized. Anesthesia was induced by placing the rat in a covered glass-container with a continuous supply of isoflurane (Isofluran Baxter, Baxter Medical, Stockholm, Sweden). After tracheostomy, the rat was connected to a ventilator and anesthesia was maintained by inhalation of 1.4–1.7 % of isoflurane in room air. End tidal pCO_2_ was monitored continuously with a capnograph (CapnoTrue AMP, bluepoint MEDICAl, GmbH, Selmsdorf, Germany). The left femoral artery was canulated for measurement of mean arterial blood pressure (MAP) and to obtain arterial blood samples used for measurement of the physiological parameters pH, pCO_2_, pO_2,_ hematocrit and electrolytes. The left femoral vein was canulated and used for infusions.

### Tissue Water Content and the permeability-surface area product (PS) for [^51^Cr]-EDTA

Change in microvascular permeability and surface area for transvascular exchange following the various treatments was investigated by measurement of the permeability-surface area product (PS) for [^51^Cr]-EDTA, as described earlier [30, 31]. For this purpose the animals received a bolus infusion of about 370 kBq of the tracer [^51^Cr]-EDTA, (0.5 mL) (GE Health Care, Stockholm, Sweden). The bolus infusion was followed by a continuous infusion of the tracer at a rate of 0.33 mL/h (3.7 MBq/mL). Arterial blood samples (10 μL) for analyses of plasma [^51^Cr]-EDTA concentration were collected at 2.5, 5, 10, 15, 25, 35 and 40 min post start of the bolus injection. After 37 min, a bolus dose of about 25 kBq of [^125^I]-albumin dissolved in 0.1 mL isotonic saline was given in the femoral vein for calculation of plasma volume in the brain. Three min later, the experiment was finished by an arterial blood sample and the animal was sacrificed by decapitation. Before each experiment [^125^I]-albumin was purified from free iodine, using centrifugal filtration. The brain and the TGs were removed and put on a chilled support. In order to analyze PS in the cortex, periaqueductal gray (PAG), trigeminal nuclei caudalis (TNC) and cerebellum, tissue structures were extracted and immediately weighed. Tissue and blood tracer activities of 51 Cr-EDTA and ^125^I-albumin were determined in a gamma counter. Tissue samples were then dried in an oven for 24 h at 100 °C and brain water content was calculated as [(wet weight-dry weight)/wet weight)] × 100. Arterial hematocrit was measured before and during tracer infusion in order to convert blood concentrations into plasma concentrations. The blood to brain transfer constant (K_i_) for [^51^Cr]-EDTA was then calculated according to the following equation K_i_ = B/ _0_∫^T^ C_a_ (t) d t [32], where B is the amount of tracer that has moved from blood to brain (tissue uptake of tracer minus regional tracer concentration in plasma), C_a_ is concentration of the tracer in arterial plasma as a function of time, and T is the duration of the experiment. K_i_ is a function of capillary plasma flow per unit mass of tissue (FV) and the permeability-surface area product (PS), the latter reflecting microvascular permeability and surface area available for diffusional exchange. The mathematical expression for this relationship is K_i_ = FV [1-e^-PS/FV^] which can be rewritten as PS = -FV ln (1-K_i_/FV) [33]. From this expression it can be deduced that with a K_i_/FV ratio of less than 0.1 the K_i_ value approximates PS with an error of less than 6 % [34].

### Evans blue

The method has recently been published; briefly 2 % Evans blue in saline was injected in the tail vein (4 ml/kg) during anaesthesia (Eftekhari et al 2015). After 30 min the animal was sacrificed and the tissues dissected out. Evans blue couple to circulating albumin and forms a large complex (EBA) which does not pass the BBB at the resting state.

### Photo microscopy

Sprague-Dawley rats (*n* = 3) were anesthetized and IS or CFA was added (see above) and monitored with a photo microscope (Olympus SZX10) at baseline and after 15 min following the addition of the inflammatory substances. For the three rats, parasagittal craniotomy, with a diameter of 6 mm, was performed and analyzed on both the left and right parietal bone.

### Statistical analysis

All data are expressed as mean ± SE, where n equals individual animals. Data were analyzed statistically using Prism 6 (GraphPad Software, Inc, CA). Differences between groups were tested using non-parametric analyzes of variance with Kruskal-Wallis test followed by Dunn’s test for multiple comparisons. Each group was compared with the vehicle group.

## Results

### Physiological data

The physiological parameters were examined after 2, 24 and 48 h for CFA, and at 2 and 24 h for IS (Table 1). No significant differences regarding measured physiological data (Na^+^, K^+^, pH, pCO_2_, pO_2_ and MAP) were observed between the groups.

**Table 1:** Physiological parameters in animals where Ki for ^51^Cr-EDTA was determined. Hematocrit in animals treated with CFA was lower than in the vehicle group at 24 h following application to the dura. No other differences regarding measured physiological data were observed between the groups

	Vehicle (*n* = 6)	CFA 2 h (*n* = 6)	CFA 24 h (*n* = 5)	CFA 48 h (*n* = 5)	IS 2 h (*n* = 6)	IS 24 h (*n* = 4)
MAP (mmHg)	98 ± 7	88 ± 3	87 ± 6	105 ± 7	85 ± 5	104 ± 9
Hct (%)	50 ± 2	46 ± 3	38 ± 2*	42 ± 1	46 ± 2	42 ± 2
pCO_2_ (kPa)	4.6 ± 0.1	4.6 ± 0.1	4.7 ± 0.2	4.5 ± 0.1	4.6 ± 0.1	4.8 ± 0.2
pO_2_ (kPa)	11.5 ± 0.1	11.6 ± 0.1	11.0 ± 0.2	12.7 ± 0.5	11.4 ± 0.3	11.4 ± 0.5
Na^+^ (mmol)	136.3 ± 0.6	138.0 ± 1.6	138.6 ± 1.0	137.0 ± 0.5	137.0 ± 0.5	136.5 ± 0.9
K^+^(mmol)	4.82 ± 0.39	4.65 ± 0.39	4.36 ± 0.13	4.40 ± 0.13	4.57 ± 0.14	4.55 ± 0.22
pH	7.50 ± 0.02	7.45 ± 0.02	7.46 ± 0.02	7.46 ± 0.01	7.48 ± 0.01	7.48 ± 0.01

Differences in plasma volumes in tissue was found following application of both CFA and IS compared to vehicle (*p < 0.05)

### Photo microscopy

**Fig. 1:**
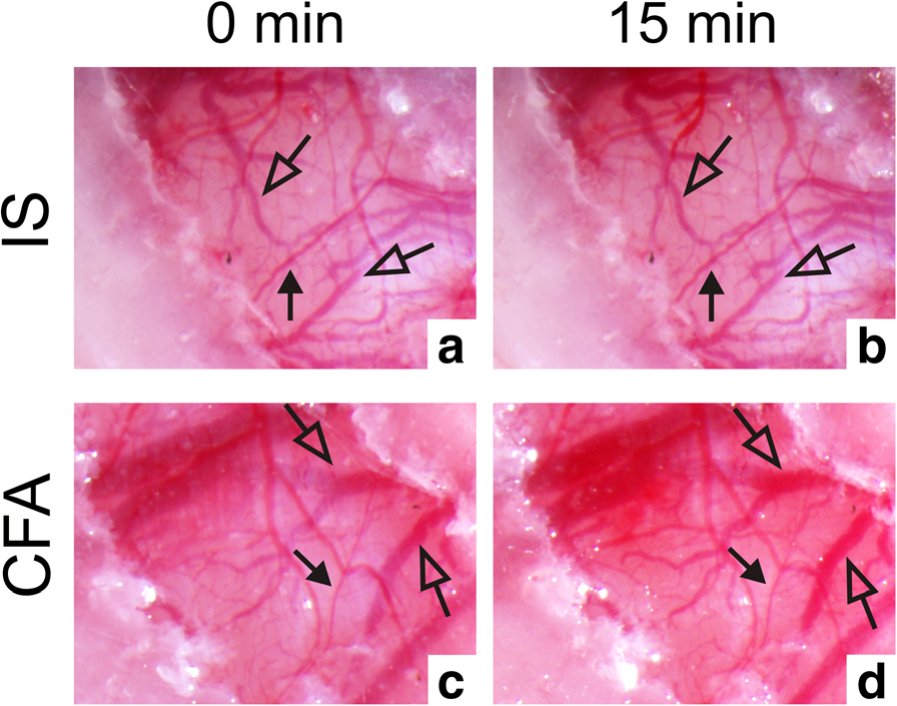
Dural effect of IS and CFA. Addition of IS and CFA on the dura mater. The control picture was taken at time 0 for IS (**a**) and CFA (**c**). The effect of 15 min. stimulation can been observed after IS (**b**) and CFA (**d**). Representative pictures. White arrows meningeal vasculature, black arrows brain vasculature

We were interested in observing whether the addition of IS or CFA in our model had any direct effect at the place of addition on the dura mater, using photo microscopy. It is worth pointing out that removal of the bone for the direct application on the dura, reduces the possible changes that can be observed in meningeal vasculature (see discussion). 15 min after the addition of IS (Fig. 1a/b) and CFA (Fig. 1c/d) we did not observe any changes in the meningeal vasculature (white arrows). However, there appears to be an weak increase in brain blood vessel diameter (black arrows), particularly after CFA.

### Permeability-surface area product for [^51^Cr]- EDTA

In order to study the permeability surface area product (PS), [^51^Cr]-EDTA was injected as a bolus dose (370 bq) followed by continuous infusion 0.33 ml/h (3.7 MBq/ml). To correct for tissue plasma volume, a bolus dose of [^125^I]-albumin was injected 3 min before the experiment was terminated and tissue collected. The plasma content, which is used to correct the [^51^Cr]-EDTA data (Table 2), was significantly higher 2 h after CFA treatment in PAG and cerebellum. 48 h after CFA treatment the plasma content was significantly higher in the spinal trigeminal nucleus. For IS a small increase was observed in the cerebellum at 24 h and with a minor change in the TNC. This increased plasma content suggests a larger blood volume that was increased due to the inflammation.

**Table 2:** Plasma volume in tissue in analyzed brain structures. Plasma volume in analyzed structures following application of CFA or IS to the dura was compared to plasma volume in analyzed structure following application of vehicle

	Control	CFA 2 h	CFA 24 h	CFA 48 h	IS 2 h	IS 24 h
Cortex (μL/mg)	7.3 + 0.6	12.2 ± 2.5	12.6 ± 0.8*	14.6 + 3.0*	11.3 ± 0.7*	11.0 ± 2.0
PAG (μL/mg)	6.0 ± 0.6	15.3 ± 3.2*	12.8 ± 1.4*	8.8 ± 1.5	13.8 ± 2.9	11.8 ± 2.3
Cerebellum (μL/mg)	9.5 ± 0.4	14.2 ± 0.8*	12.8 ± 0.5	29.4 ± 6.2 *	13.8 ± 1.7	16.3 ± 1.3*
L Spinal Trigeminal nucleus (μL/mg)	10.3 ± 1.7	14.3 ± 2.3	21.2 ± 2.1*	44.0 ± 14.4*	12.5 ± 3.5*	27.0 ± 8.9
R Spinal Trigeminal nucleus (μL/mg)	12.8 ± 2.6	13.7 ± 1.6	19.4 ± 1.8	23.6 ± 3.4*	16.0 ± 1.8	28.0 ± 11.0
L Trigeminal Ganglion (μL/mg)	52.3 ± 4.3	53.3 ± 12.5	48.0 ± 8.0	25.8 ± 3.1	40.5 ± 13.4	59.3 ± 10.3
R Trigeminal Ganglion (μL/min/g)	54.5 ± 5.7	51.7 ± 10.4	68.2 ± 13.9	22.0 ± 2.6	56.7 ± 15.5	83.8 ± 27.4

Differences in plasma volumes in tissue was found following application of both CFA and IS compared to vehicle (*p < 0.05)

**Fig. 2:**
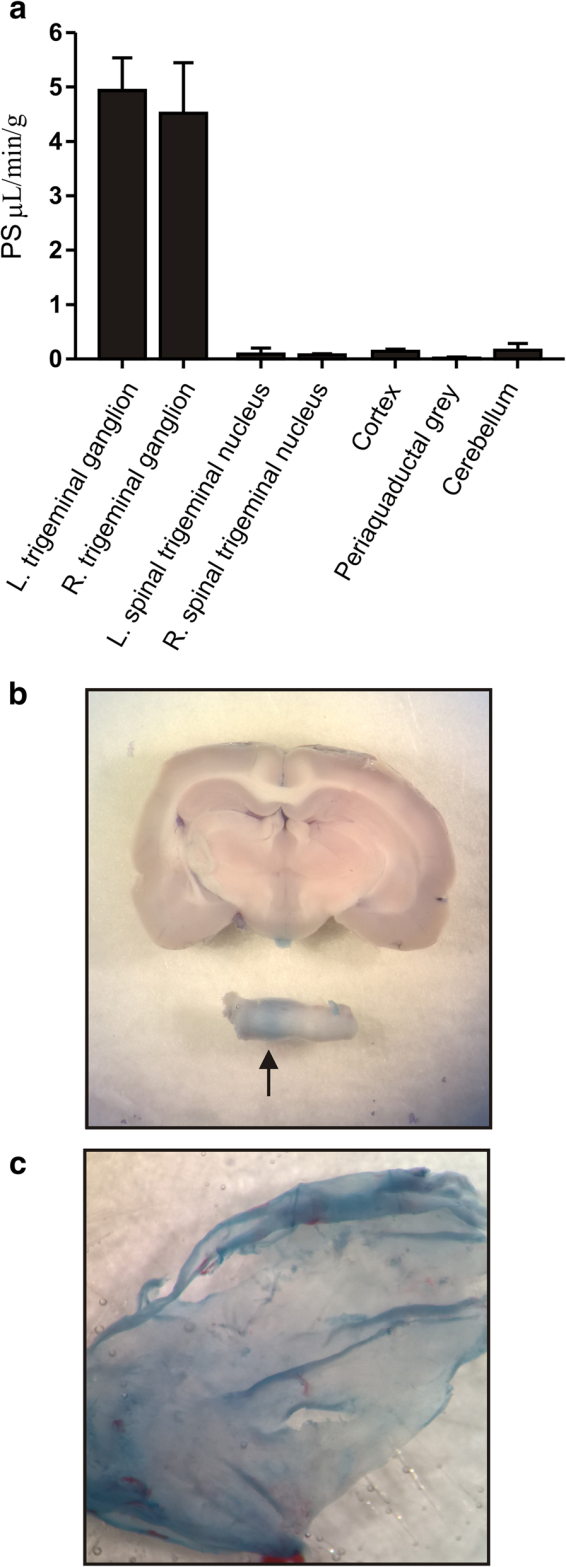
Comparison of the PS for the different structures analysed. **a** The PS of the TG is much larger than that of the other structures investigated. **b** Rat brain and TG after perfusion of Evans blue shows that the TG is permeable whereas the brain is not. **c** The dura mater is permeable to Evans blue

We investigated the left and right TG and TNC individually. IS and CFA was only added on the left parietal bone, and we were interested in observing a direct link between the dural application and the corresponding TG and TNC structures. The control data where saline has been applied to the dura mater show that the TG is >30 times more permeable than cerebellum/cortex and >200 times than the PAG (Fig. 2a). In addition, the permeability of the TG was also >30 times higher than the spinal TNC. This clearly illustrates that the TG has much higher permeability than the CNS and is also in agreement with the 3 % of triptans/gepants that can reach the CNS [5, 9–11] as the cortex/cerebellum has a permeability that is ~3 % of TG. In addition we also show that Evans blue, that cannot pass the BBB (Evans blue binds to serum albumin, creating a large molecule that can be visualized), colours the TG but not the brain (Fig. 2b). The dura mater was likewise “blue” after the administration of EBA (Fig. 2c). Due to the operation and manipulations we did not quantify the dura mater permeability.

**Fig. 3:**
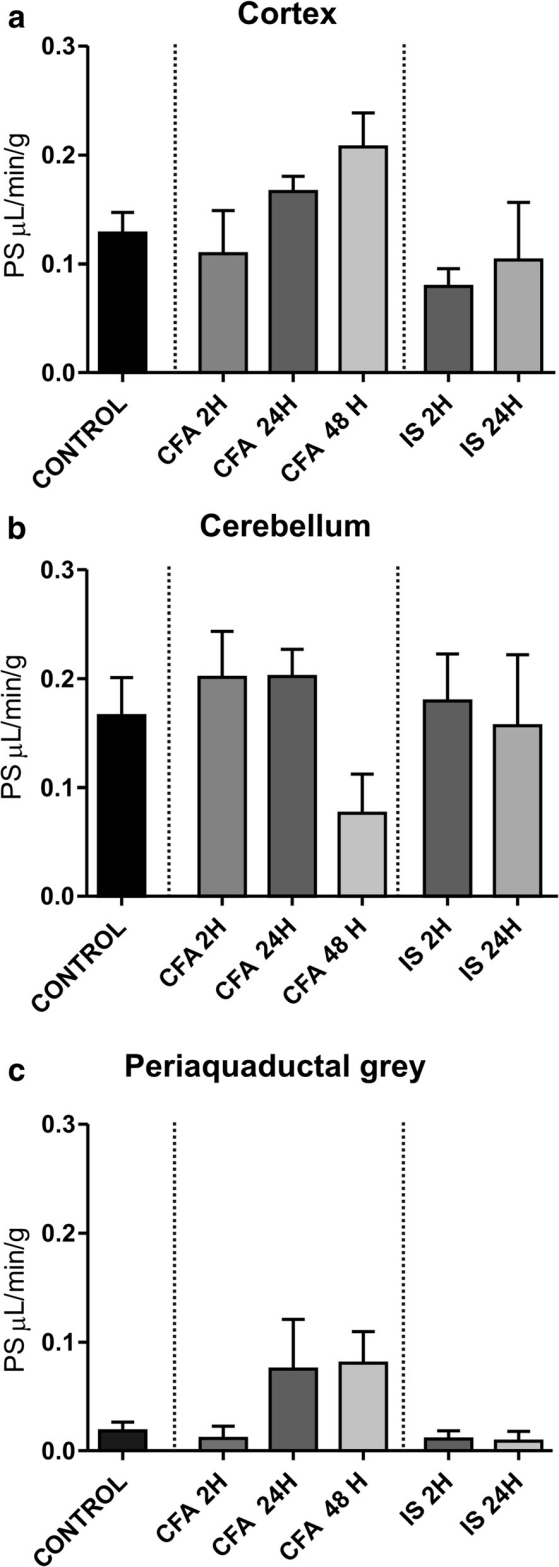
PS in the cortex cerebellum and PAG after treatment with CFA or IS. The figure shows PS for ^51^Cr-EDTA in the cortex (**a**), cerebellum (**b**) and periaquaductal grey (**c**). PS for treatment following application of CFA or IS to the dura was compared to PS following application of vehicle to the dura. *p ≤0.05

**Fig. 4:**
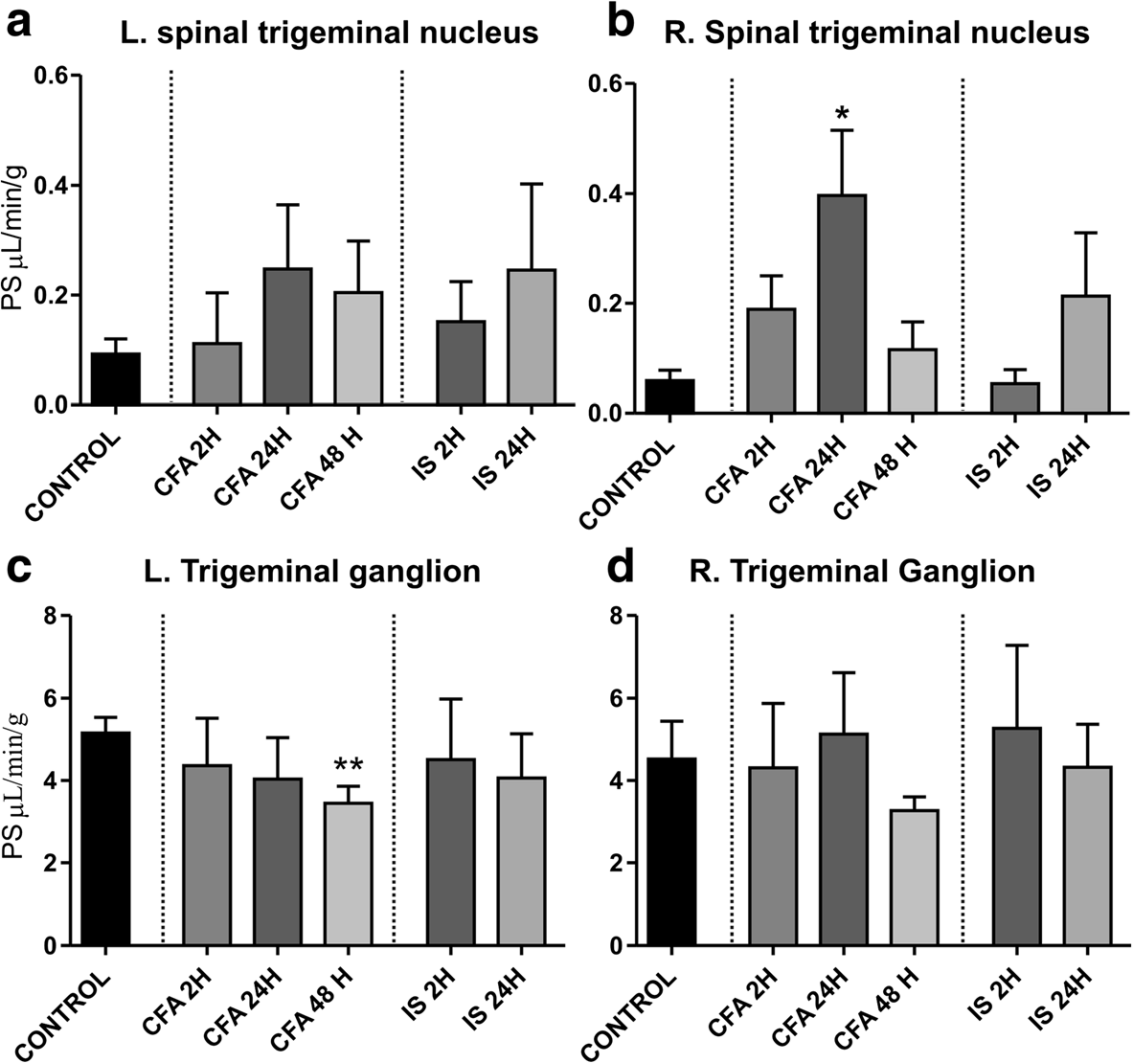
PS in the TG and spinal trigeminal nucleus after treatment with CFA or IS. The figure shows PS for ^51^Cr-EDTA in the left TNC (**a**), right TNC (**b**), left TG (**c**), and right TG (**d**). PS for treatment following application of CFA or IS to the dura was compared to PS following application of vehicle to the dura. *p ≤0.05

We further characterized the changes in permeability following the addition of CFA and IS onto the dura. There were no significant increases in the permeability of the cortex, cerebellum or PAG following either of the inflammation triggers (Fig. 3) compared to vehicle, suggesting that permeability does not change in the inflammatory models. A small permeability increase in the spinal TNC was observed after 24 h of CFA, although the change is nearly negligible as it after CFA still is >10 times less permeable than the TG (Fig. 4). We observed a small decrease in the left TG with the same tendency in the right TG, which could be due to the increased ganglion activation. We did not observe any significant changes in any of the structures examined after either 2 or 24 h after the addition of IS.

To ensure that there was no oedema in the brain, we measured the brain water content (Additional file 1: Figure S1). There were no significant changes in water content in the CNS. Only the left TG showed an increase of 10 % at 48 h after CFA application onto the dura. This also supports the permeability studies as a lowered permeability in the BBB, most likely would have led to increased water content.

## Discussion

This is the first study designed to quantify and compare the permeability in the TG and the CNS. We present results that show the PS (and hence the permeability) was more than 30 times higher in the TG than in other structures (CNS) analysed, strongly indicating that the TG is indeed outside the BBB. The addition of the inflammatory agents, IS or CFA, did not change the permeability in the CNS structures, despite demonstrating an inflammatory reaction in the TG [24]. This will be discussed below in the relation to the pharmacokinetics/BBB and the TG as a possible target for acute migraine treatment. The dura mater does not have a barrier to EBA (Fig. 2c), since we operated in this area it was not included in the quantification. It is worth noting that it is the part of TG that contains the neurons that are “blue” in the TG and that the pale part is the trigeminal nerves (Fig. 2b).

## Changes in plasma content

Tissue plasma volume was determined by measured radioactivity of ^125^I, which represents radioactivity from only plasma. Leakage of infused radioactive albumin during the 3 min of its circulation time must be regarded as insignificant, and has been analysed before [35]. This means that the calculated increase in plasma volume, most likely, was an effect of inflammatory-induced vasodilation rather than extravasation. In addition, we observed no changes in brain water content which illustrates that there is not a large extravasation of water and that the intracranial pressure is maintained after the addition of either of the inflammatory substances. It is worth noting that an increase in plasma volumes after application of CFA and IS may affect the calculations of PS, especially in areas with low permeability for [^51^Cr]-EDTA in terms of in underestimation of PS (Ki), as B is indirectly dependent on plasma volume (see Methods), The theory behind the present method has been discussed before, see Chodobski and colleagues [14].

## Permeability of TG/CNS in relation to BBB

The results presented here, show that permeability was significantly higher in the TG than in other structures analysed (Fig. 2), both after addition of vehicle and after application of CFA or IS. This illustrates that the drug-availability will be much higher in the TG compared to the CNS and supports this as a potential target for anti-migraine drugs [9]. The inflammatory model has been utilized in several studies and show similar findings to migraine for example TG activation with an increase in p-ERK1/2 activation and IL-1β expression, hypersensitivity to mechanical and thermal stimulation, as well as activation in the TG and in the brain stem trigeminal neurons, similar to what could be expected in migraine [17, 24, 29, 36, 37]. Since it has been suggested that the BBB in migraine patients might be defective or transiently altered [12], we tested if the BBB was altered in the present method. Obviously this is not a “true” migraine model; it may be regarded as a surrogate method. IS has been used in many studies for the understanding of for example trigeminal involved sensitization [17]. CFA is a well-known method to induce local inflammation and we postulate that this may share aspects with the more chronic forms of migraine. In relation to other models, there exists one study on repeated cortical spreading depression and the BBB in rats [25]. Here a mild BBB breakdown/leakage was observed including brain edema and protein leakage. However, the cortical depression has to be very strong for these effects, for example after cerebral ischemia where there is a strong cortical spreading depression, it still takes several hours for the BBB to be broken down [38]. In addition, there are no clear proof of breakdown or leakage in the BBB in patients during migraine attacks [8]

Interestingly, in a study by Markowitz and colleagues, extravasated ^125^I-BSA or Evans blue was noted in the dura of saline-perfused rats following intravenous capsaicin or following electrical stimulation of the rat TG. Extravasation was however not observed in the brain following chemical, electrical, or immunological stimulation nor following topical application of capsaicin to the pial surface or injection into the cortex of rats. The data are therefore showing similar indication as us, with our inflammatory model [39]. As we did not observe any alteration in the BBB, we add our data to the negative experiments that the BBB does not change its permeability during migraine attacks. Our results are in agreement with clinical data observations [26–28].

## Direct effects on vasculature upon the addition of CFA and IS

The addition of the inflammatory substances onto the dura mater raises an interesting question in relation to the vasogenic theory. Does the inflammatory substance lead to vasodilation? IS caused a mild contraction and CFA had no vasomotor effect per se on the in vitro MMA, studied using wire myography [24]. In our model, we could however not observe any changes in the meningeal arteries. Interestingly, Williamson and colleagues showed that in closed cranial window an increase in the MMA diameter of up to 180 % could be seen with the addition of CGRP [40]. However, in open cranial window only 15 % was observed [41], and therefore changes are difficult to detect. The effect of IS and CFA on the MMA will require further analysis, but this is not in the scope of this study. Here we show that in our model of application of IS and CFA we do not see any changes in MMA, we do however see an increased blood flow to this area, particularly for CFA.

## Conclusion

In conclusion, we observed that [^51^Cr]-EDTA does not pass to the CNS in a major way. However, [^51^Cr]-EDTA readily passed the TG by >30 times compared to the CNS. Application of CFA or IS (studied at 2, 24 or 48 h after the application) did not show altered transfer constant to [^51^Cr]-EDTA. Thus, with these experiments we suggest; (i) dural triggered TG inflammation, with IS or CFA does not change BBB passage, and (ii) the TG is readily exposed to circulating molecules and could provide a site where anti-migraine drugs could interact with the trigeminal system.

## Additional file

Additional file 1: Figure S1. Analysis of tissue water content. Tissue water content in the investigated tissue structure of the brain and in the trigeminal ganglia is shown. Tissue water content following application of CFA or IS to the dura was compared to brain water content following application of vehicle to the dura. **p* ≤0.05. (TIFF 515 kb)

## Abbreviations

BBB: Blood - Brain Barrier
CFA: Complete Freund’s Adjuvant
CGRP: Calcitonin Gene-Related Peptide
CNS: Central Nervous System
EBA: Evans Blue Albumin
IL: InterLeukin
IS: Inflammatory soup
MAP: Mean Arterial blood Pressure
MMA: Middle Meningeal artery
PAG: PeriAqueductal Gray
PS: Permeability Surface area product
TG: Trigeminal Ganglion
TNC: Trigeminal Nucleus Caudalis
