# Supplementary figures and images for: Experimental inflammation following dural application of complete Freund’s adjuvant or inflammatory soup does not alter brain and trigeminal microvascular passage
Source: J Headache Pain. 2015 Oct 28;16:91. doi: 10.1186/s10194-015-0575-8 (PMC4627622; doi:10.1186/s10194-015-0575-8)

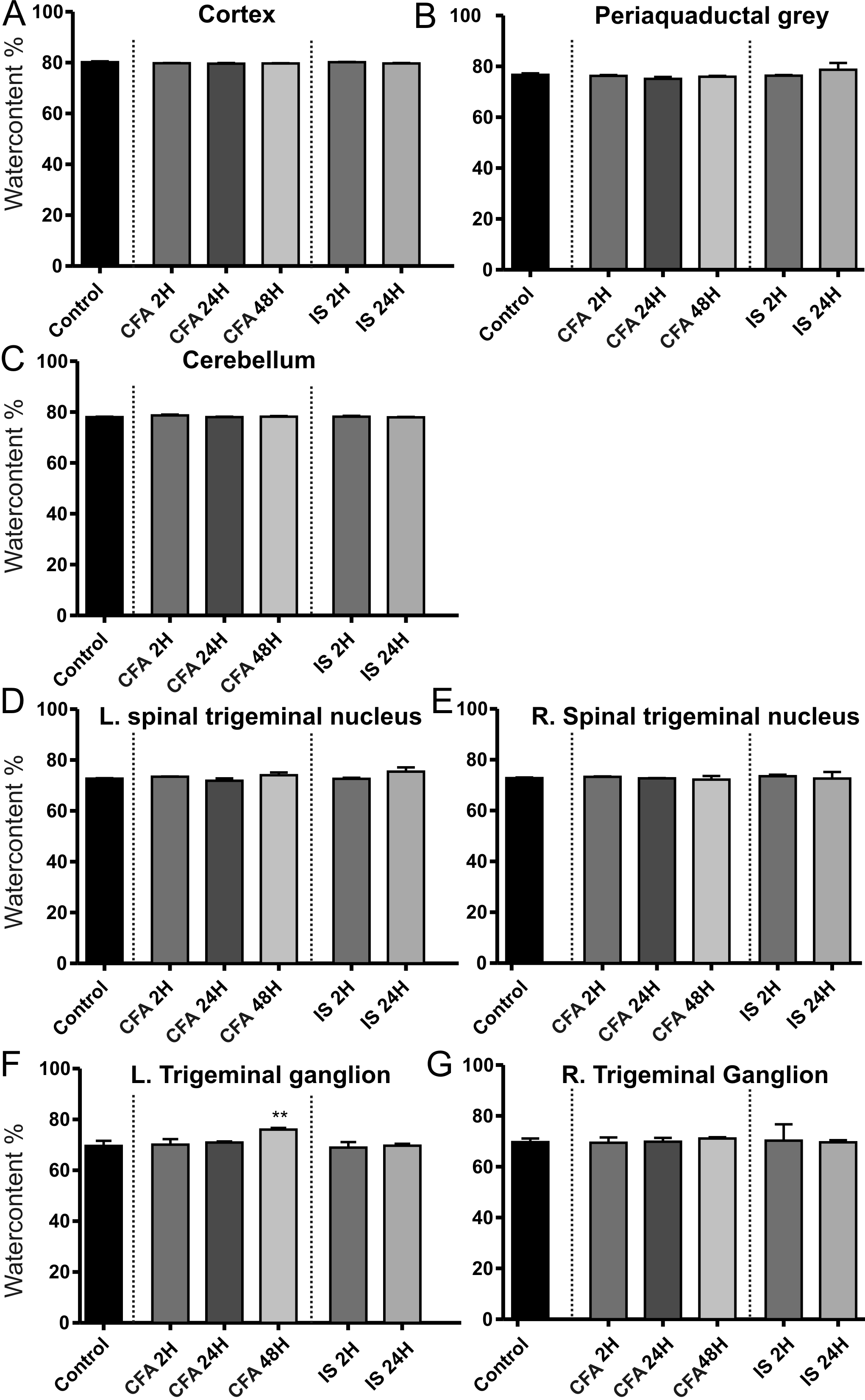

Supplement: Additional file 1: Figure S1. — Analysis of tissue water content. Tissue water content in the investigated tissue structure of the brain and in the trigeminal ganglia is shown. Tissue water content following application of CFA or IS to the dura was compared to brain water content following application of vehicle to the dura. *p ≤0.05. (TIFF 515 kb) [file 10194_2015_575_MOESM1_ESM.tif]
